# Assessment of fluid status in neonatal dialysis: the need for new tools

**DOI:** 10.1007/s00467-022-05829-2

**Published:** 2022-12-02

**Authors:** Noureddin Nourbakhsh, Nadine Benador

**Affiliations:** 1 Pediatric Nephrology, University of California San Diego, La Jolla, CA USA; 2 Rady Children’s Hospital, San Diego, CA USA

**Keywords:** Dialysis, Fluid overload, Fluid balance, Hypertension, Children, Pediatric

## Abstract

**Background:**

Assessment of fluid status in neonatal dialysis has largely focused on traditional tools including clinical assessment, serial weights, and blood pressure (BP) measurements. However, in infants on kidney replacement therapy, the assessment of fluid overload is problematic due to errors in weight assessment, subtlety of physical exam findings, and inaccuracy of non-invasive BP measurements.

**Case summary:**

In this presentation of a neonate with bilateral renal agenesis requiring kidney replacement therapy, the treating team assessed a number of variables in determining the ultrafiltration prescription for dialysis across 2 modalities (hemodialysis and continuous kidney replacement therapy).

**Complications:**

Fluid overload, cardiomegaly, and worsened respiratory status occurred when attempting to assess the neonate’s fluid status by traditional markers (weights, blood pressures, physical exam findings). B-type natriuretic peptide (BNP) was obtained and was noted to correlate with the degree of fluid overload.

**Key management points:**

Compared to traditional tools for assessment of fluid status in pediatric dialysis, BNP assisted the medical team in optimizing the volume status of the subject and determining optimal daily ultrafiltration goals. Due to the rapid release in response to myocardial stretch and the lack of kidney clearance of the peptide, BNP may represent an objective, timely, and reliable index of volume status in the pediatric dialysis patient.

## Case part 1

A baby girl was born via C-section at 37-week gestation. Birth weight was 2.19 kg. She is the product of an IVF pregnancy, donor egg and sperm, born to a 43-year-old G3P0 with 2 prior miscarriages. At 18-week gestation, it was discovered that the baby had bilateral renal agenesis. Mother had chorionic villus sampling at 20-week gestation, which revealed normal karyotype XX. She was treated with serial transcervical amnio-infusions (twice weekly) to minimize pulmonary hypoplasia.

The mother’s past medical history is significant for MTHFR mutation and Factor V Leiden. She was on aspirin and enoxaparin during the pregnancy. She was admitted to an outside hospital after a failed amniotic infusion. There was an amnion chorion separation that day that prevented further attempts at saline infusions. Even with small contractions, the baby had variable decelerations to as low as 50 beats per minute. C-section took place, and the baby was born vigorous with Apgar scores of 8 and 9. She required only transient blow-by oxygen and was transferred to the NICU. She remained on room air, with peripheral intravenous catheter in place.

On day of life (DOL) 3, peritoneal dialysis (PD) catheter and gastrostomy tube were placed. Patient received intravenous cefazolin prophylaxis in the operating room. Two days later, vancomycin was initiated due to increased erythema around surgical incision site. Site culture was negative. Four days after PD catheter placement, the patient was started on hourly low volume PD exchanges, continuously over 24 h. Fill volume was initially 10 mL (4.5 mL/kg) for 6 exchanges, then increased and maintained at 15 mL (6.8 mL/kg). Dialysate used was 2.5% dextrose solution medicated with heparin 500 units/L. Despite this low fill volume, the patient developed a dialysate leak at the catheter site at 10 days of age. Hourly PD exchanges were held and PD catheter was locked with heparin. An 8 Fr by 18 cm left internal jugular tunneled hemodialysis catheter was placed, and dialysis modality was changed to daily 3-h hemodialysis (HD) sessions using a low flux polysulfone dialyzer membrane (Hemoflow F3 — Fresenius). The circuit, including the filter and tubing, had a volume of 47 mL and a blood prime was used. Dialysis prescription consisted of a blood flow rate of 14 mL/min (6.4 mL/kg/min), a dialysate flow rate of 300 mL/min, and net ultrafiltration (UF) obtained was between 100 and 160 mL per treatment (17 to 23 mL/kg/h or 50–70 mL/kg per session), adjusted daily based on traditional volume assessment parameters such as blood pressure, weight change, and evaluation of intake/output.

**Fig. 1:**
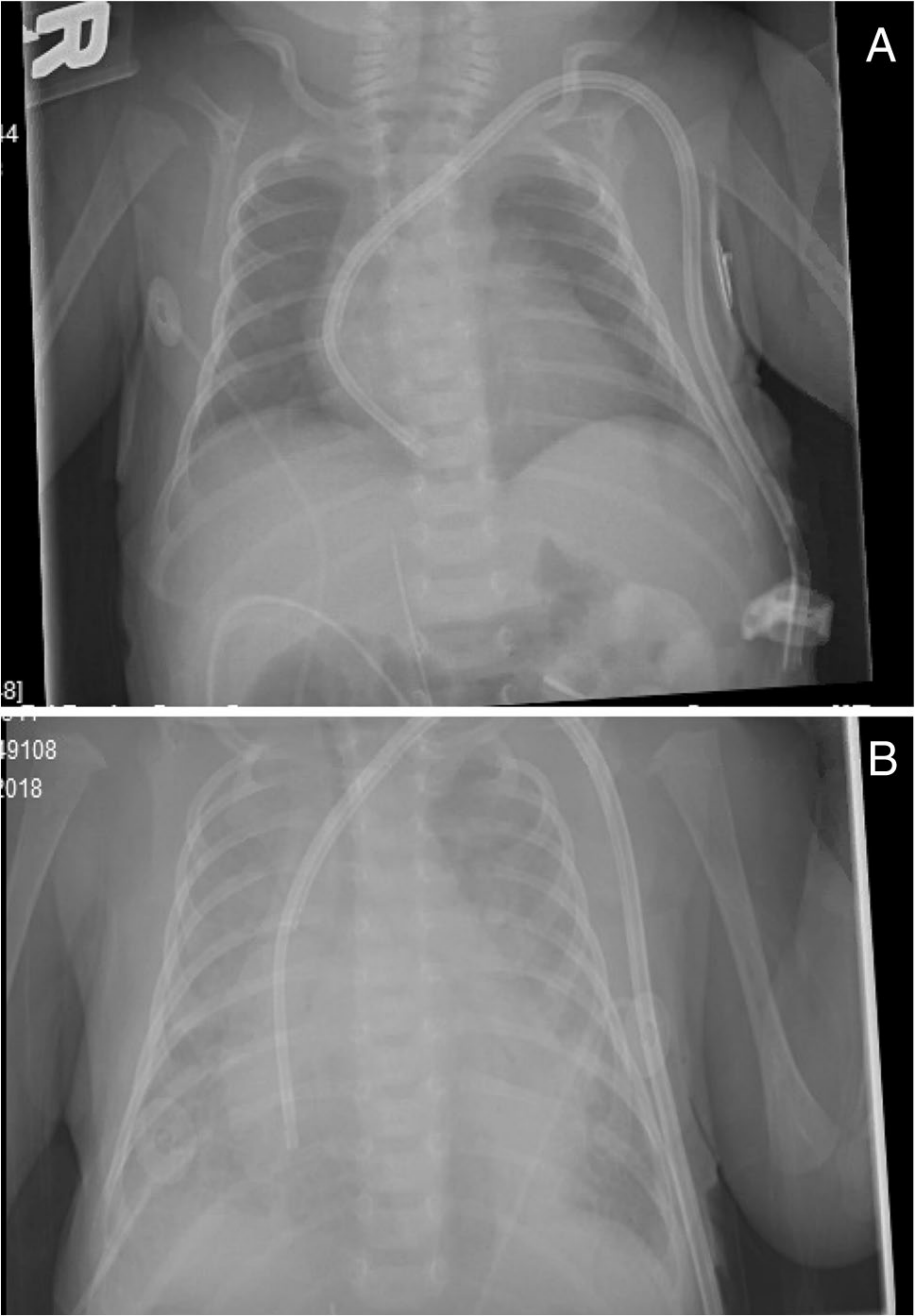
**A**, **B** Chest x-ray of infant born with bilateral renal agenesis on day of life 8 (**A**). Chest x-ray of infant at 3 weeks of age in respiratory distress (**B**)

The patient’s weight at time of initiation of HD was 2.5 kg; however, dry weight was estimated to be 2.2 kg. After 6 days of daily HD, patient had achieved a weight of 2.36 kg, suggesting improvement in fluid balance. Intake/output balance was positive 50–100 mL/day (21–41 mL/kg/day). Total fluid intake was increased to 100 mL/kg/day on DOL 15 and increased further to 120 mL/kg/day on DOL 17. On DOL 18, PD was reattempted; however, catheter had inflow obstruction and the PD nurse was unable to infuse dialysate. After manipulation with manual flush, low volume PD exchanges with the goal to maintain patency of the catheter were resumed (10 mL fill volume = 4.5 mL/kg). While on these hourly PD minimum exchanges, daily HD was continued. The daily UF obtained with PD was 40–50 mL/day. On DOL 19, UF goal for HD was increased to 75 mL/kg as the patient’s pre-HD weight was increasing too fast, now 2.68 kg. On DOL 20, pre-HD weight was 2.62 kg despite UF the day before of 80 mL/kg and net fluid balance of + 75 mL. UF goal was increased further to 85 mL/kg for HD treatment on DOL 20. Of note, pre-HD BP was higher at 104/62 prior to HD on DOL 20. PD catheter leak recurred so PD exchanges were discontinued. At 3 weeks of age (DOL 21), the patient had increased work of breathing and required intubation. Chest x-ray was performed and revealed bilateral pulmonary edema and an enlarged cardiac silhouette (Fig. 1A and B). As her respiratory status worsened, she required intubation. Within 2 h of intubation, she was converted from conventional ventilation to high-frequency ventilation.

Differential diagnosis for patient’s acute decompensation included:

- Septic shock
- Respiratory event (pneumonia, mucous plugging, pneumothorax)
- Acute decompensated heart failure
- Volume overload

## Case part 2

**Fig. 2:**
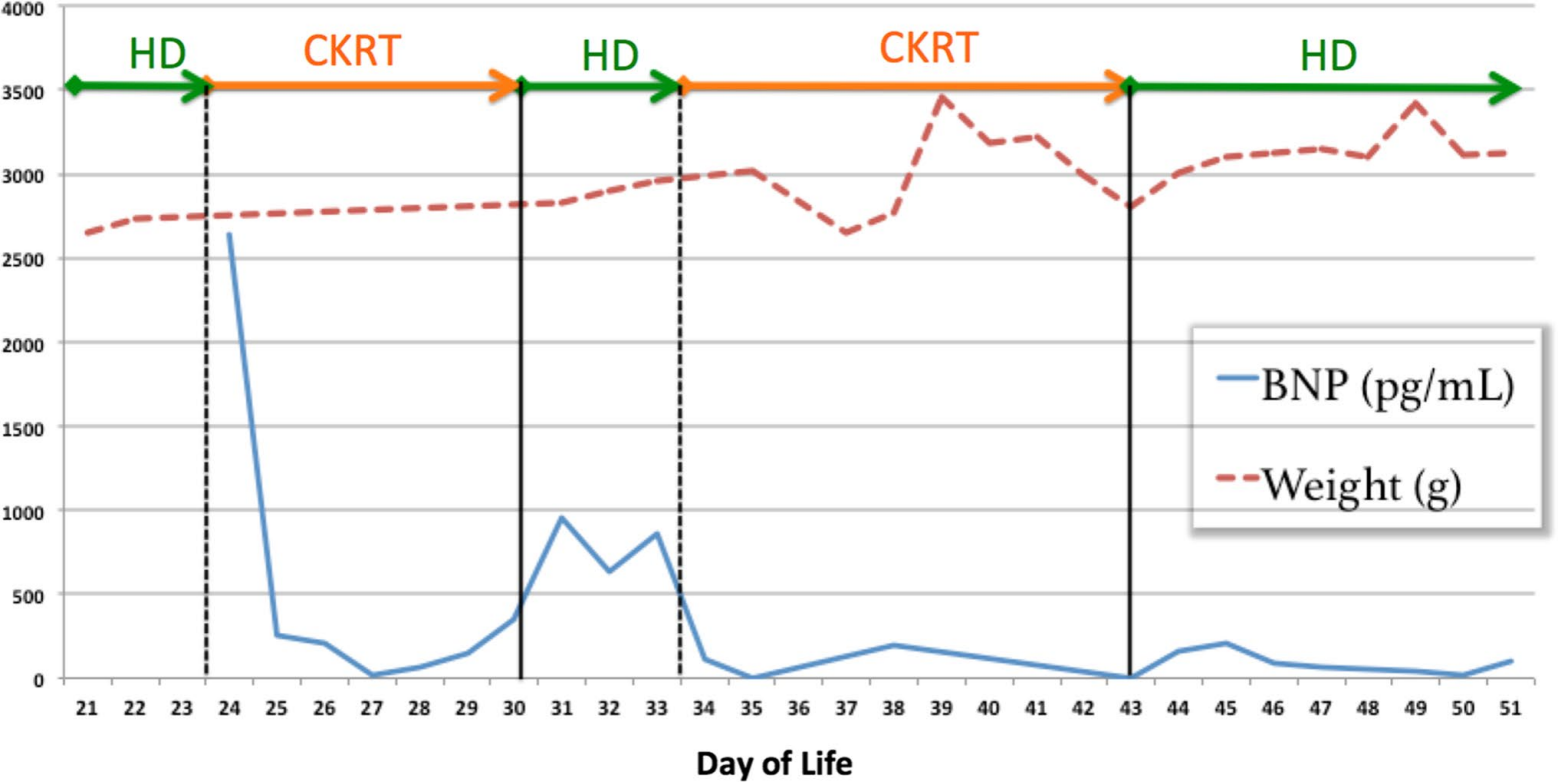
Linear plot of values comparing BNP and weight with respect to KRT modality used

The patient had minimal oxygen requirement prior to intubation (oxygen given via nasal cannula). Her chest x-ray following intubation showed signs of volume overload (enlarged cardiac silhouette), but no evidence of pneumothorax or pneumonia. Prior to her decompensation, net ultrafiltration of 220–230 mL/day was obtained with daily intermittent hemodialysis. Her average blood pressure was 91/47 prior to her respiratory event. Blood cultures were drawn including from HD catheter; patient had already been continued on vancomycin for surgical site cellulitis. Vancomycin levels were at goal. Meropenem was added to broaden coverage. Blood cultures were found to be negative after 48 h and antibiotics were discontinued. The patient’s prior echocardiogram showed normal LV function, patent foramen ovale with left to right shunting, and a bicuspid aortic valve. Echocardiogram on the day of respiratory failure showed decreased LV systolic shortening and an ejection fraction of 35%. The NICU team started epinephrine and nitroprusside IV continuous infusion. B-type natriuretic peptide (BNP) was ordered due to the enlarged cardiac silhouette and value was found to be > 5000 pg/mL. Dialysis modality was changed to continuous veno-venous hemodiafiltration (CVVHDF) with Prismaflex HF1000 polyarylethysulfone high flux dialyzer membrane. CVVHDF was conducted with normalized blood prime, blood flow rate of 20 mL/min (8 mL/kg/min), dialysate flow rate of 200 mL/h (83 mL/kg/h), replacement fluid of 100 mL/h, and regional citrate anticoagulation. Net UF of 80 mL was performed in the first hour, followed by UF rate of 10 mL/h (4 mL/kg/h) thereafter. Calcium-free dialysate (Prismasate 4/0/1.2) with added phosphorus and magnesium was utilized. After 48 h of CCVHDF, UF goal was changed to even. BNP values normalized after 4 days of CVVHDF but then increased mildly again prior to transitioning back to daily HD 2 days later (Fig. 2). Normal BNP value was considered to be less than 34 pg/mL based on study data from healthy newborns [1].

Without use of BNP to guide UF goals, the patient developed fluid overload again and required placement back on CVVHDF. After the BNP levels normalized one more time while on CVVHDF, she was converted back to intermittent daily hemodialysis. We started measuring BNP pre- and post-dialysis treatment and employing this technique as a quantitative marker of fluid overload, were able to adjust the UF goal to keep the patient with a normal BNP post-dialysis.

For example, we noticed that with a pre-dialysis BNP of 90 to 150 pg/mL, a net UF per treatment of around 200 mL (91 mL/kg) led to a normal post-dialysis BNP. The net UF goal was adjusted between 170 mL (77 mL/kg) and 300 mL (136 mL/kg) based on the pre-dialysis BNP. Sometimes, dialysis treatment time was increased by 30 min to attempt to reach the higher goal.

The patient’s cardiac output and respiratory status improved. She was extubated within 1 week. The child had no further episodes of fluid overload and remained on daily HD. Another benefit of transitioning from CKRT to daily HD once optimal fluid status was achieved was the ability for increasing the patient’s physical activity and parental bonding which were more difficult to achieve previously while patient was connected to the dialysis circuit for 24 h a day. Patient was able to transition back to PD when exit site had healed sufficiently and she eventually was treated exclusively with PD by 3 months of age.

## Background

B-type natriuretic peptide (BNP) was first isolated from porcine brain in 1988 and found to be synthesized and secreted from the ventricular myocardium [2]. The main source of BNP secretion from the ventricular myocardium is via rapid gene expression with de novo synthesis of the peptide [3]. The stimulus for BNP secretion was elegantly elucidated by Yasue et al. in 1994 [4] with 2 major conclusions: firstly, the secretion of BNP from the left ventricle (LV) increases in proportion to the severity of LV dysfunction and secondly, plasma levels of BNP reflect the LV secretion rate of these hormones and may be used as surrogate markers of left ventricular dysfunction [4]. As clinical assays for heart failure and LV dysfunction were developed, two distinct markers of ventricular wall stretch arose: N-terminal pro-BNP (NT-proBNP, the N-terminal fragment of B-type natriuretic peptide) and BNP. In patients with kidney disease, BNP has the advantage over NT-proBNP in that the former is not cleared primarily by kidney excretion and has a shorter half-life [3]. In the general adult population, BNP and NT-proBNP levels were both found to be elevated in distinct clinical settings compared to baseline: older age [5], anemia [6], presence of left ventricular hypertrophy [7], coronary artery disease [8], malnourished state [9], and sepsis [10].

Given the rapid synthesis and release of BNP in response to myocardial stretch, the potential for measurable assessment via peripheral blood sampling, and the lack of kidney clearance of the peptide, BNP became a marker of interest in the assessment of volume overload in adult hemodialysis patients. In the adult literature, elevated natriuretic peptide values correlate with increased risk of cardiovascular and all-cause mortality in the kidney failure population [11]. Fewer studies exist which evaluate the association between BNP and extracellular water in hemodialysis patients. One such study by Fagugli et al. examined the association between BNP and extracellular water in hemodialysis patients and found that extracellular water was the strongest independent variable predictive of release of BNP from the left ventricle [12].

In summary, there appears to exist a strong correlation between volume overload and BNP in adult patients with CKD stage 5 on dialysis; however, the use of BNP in this population has been limited to prognostication of cardiovascular events rather than as an assessment of volume status, likely due to presence of confounding variables in the adult population.

## Pediatric implications

To assess fluid overload in children on dialysis, traditional tools include clinical assessment, serial weights, and blood pressure measurements. Optimizing intravascular volume is crucial for these patients as, similar to the adult literature, cardiovascular complications are the most common cause of mortality in children [13]. In infants and young children on kidney replacement therapy, the assessment of fluid overload is rendered even more problematic given that the evaluation of dry weight may be mistaken for appropriate nutritional weight gain. The examiner may not be able to readily identify typical physical exam findings of fluid overload. Non-invasive BP measurements may be inaccurate due to patient cooperation and there exists a subset of pediatric patients on dialysis with low blood pressures at baseline. While other diagnostic instruments for evaluation of fluid overload are under development, including lung ultrasound, inferior vena cava collapsibility index, and bioimpedance [14], these tools have not been validated nor are they readily accessible during routine clinic visits.

There are few studies regarding BNP in children. The question of normative values of BNP in neonates was evaluated by Rodriguez et al. and found to be 5–34 pg/mL in healthy newborns [1]. Zoair et al. evaluated the relationship between NT-proBNP and LV dysfunction in thirty pediatric hemodialysis patients compared to age-matched controls. In this study, there was a negative linear correlation between ejection fraction and NT-proBNP [15]. In another study by Nalcacioglu et al., NT-proBNP was evaluated as a marker for body fluid status in children with CKD and found to be a promising diagnostic and prognostic tool [16].

With respect to blood pressure variation, in the largest study of pediatric peritoneal dialysis patients with hypertension, change in BNP correlated strongly with change in systolic blood pressure and change in diastolic blood pressure [17].

As mentioned for the adult population, due to the rapid synthesis and release of BNP in response to myocardial stretch, and the lack of kidney clearance of the peptide, BNP may represent an objective and reliable index of volume status in the pediatric dialysis patient. Additionally, the lack of confounding variables (LVH, advanced age, coronary artery disease) in the pediatric population make BNP an appealing marker of volume overload in children on dialysis.

## Discussion

**Fig. 3:**
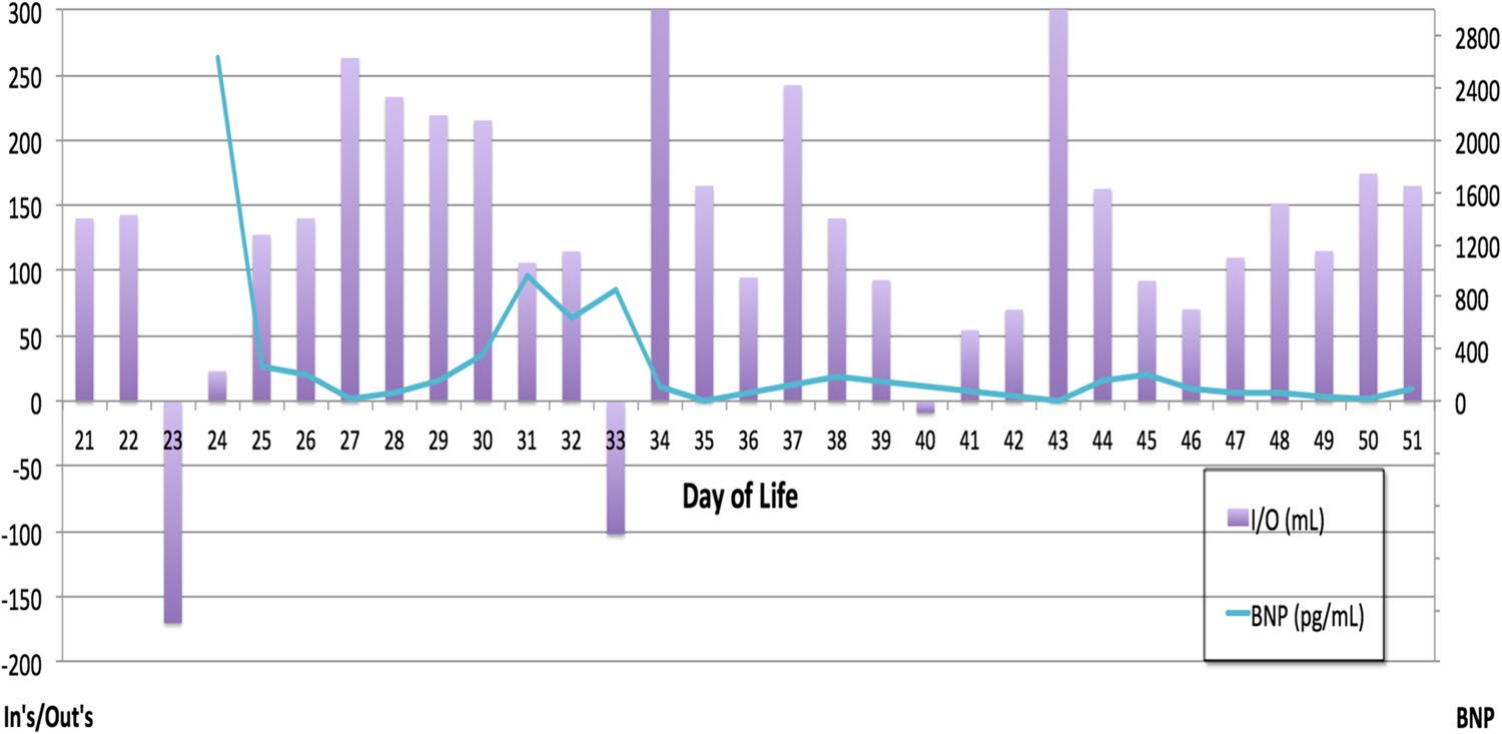
Linear plot values of BNP and in’s/out’s over same time period as Fig. 2

**Fig. 4:**
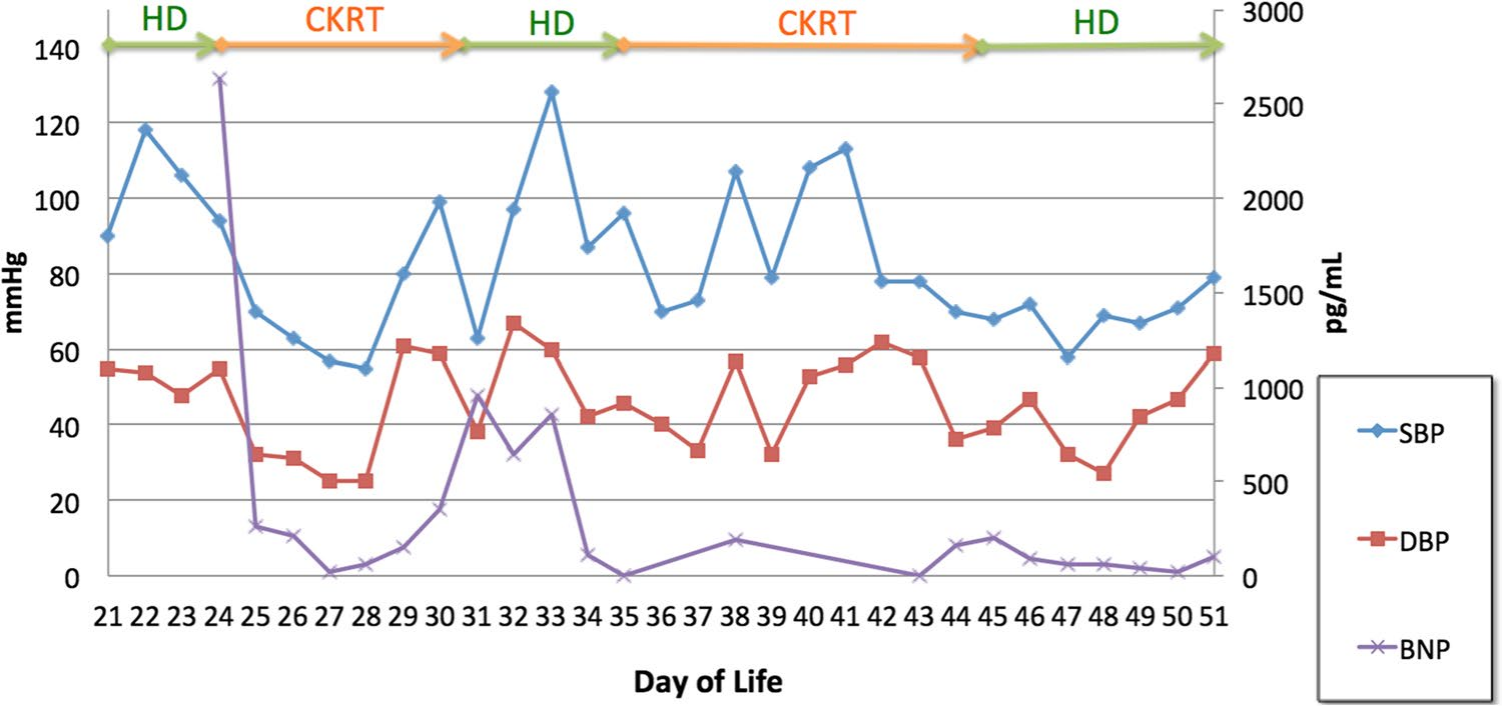
Linear plot of values comparing BNP with average SBP and DBP. KRT modality used is also noted above plot

With respect to traditional markers of volume status in pediatric dialysis, we compared our patient’s BNP to daily weight assessment (Fig. 2), daily intake/output (Fig. 3), and average daily blood pressures which were obtained by oscillometric measurements (Fig. 4). Of note, BNP exhibited daily variation while the subject’s weight remained flat at a time of fluid overload (day of life 22 to 32 on Fig. 2), suggesting alterations in ventricular myocardial stretch and that BNP may be more reflective of volume status rather than weight measurements taken on a bed scale which may be inaccurate. Figure 3 is notable for significant discrepancies between intake/output balance of the child and BNP values. For example, on DOL 23, the patient had her largest negative fluid balance and the following day (DOL 24) had a severely elevated BNP. Additionally, on DOL 34, the patient had a large positive fluid balance but declining BNP value. Figure 4 highlights the correlation between blood pressure and BNP. SBP correlated better with BNP values compared to DBP. As is well known in pediatric practice, BP is often difficult to obtain accurately and confounded by many variants such as awake state, crying, and feeds.

Lastly, Table 1 shows the correlation between echocardiographic findings and BNP values in this case. Similar to the echocardiographic findings described in the Zoair study [15], when the patient’s BNP was greater than 5000 pg/mL and she developed cardiomegaly and respiratory distress, her echocardiogram was notable for severe left atrial hypertension, severely diminished left ventricular systolic shortening, and an ejection fraction of 35%. In this case, LV systolic dysfunction correlated most closely with elevations in BNP, while BNP values of less than 1000 pg/mL resulted in echocardiographic findings of normal LV function (Table 1).

**Table 1:** BNP values and echocardiogram findings

	DOL 23	DOL 24	DOL 25	DOL 27	DOL 31	DOL 33	DOL 35	DOL 45
BNP (pg/mL)	> 5000	2639	259	21	959	857	< 10	204
LV	Decreased LV systolic shortening	Severely reduced LV systolic function	Mild LVH; underfilled LV with normal systolic function	Normal LV size and normal systolic shortening	Normal LV size and normal systolic shortening	No change from prior echo	No change from prior echo	No change from prior echo
LA	Severe LA hypertension	3 mmHg mean gradient through the PFO	Small PFO with unrestrictive left to right flow. Mildly dilated left atrium	No changes	PFO, small shunt	No change from prior echo	No change from prior echo	No change from prior echo
EF	35%	40%	63%	68%	63%	70.1%	65%	67%
KRT modality	HD	CKRT	CKRT	CKRT	HD	HD	CKRT	HD

In our case presentation, BNP levels provided an additional objective tool to use with traditional important markers of fluid status such as weight, blood pressure, and fluid balance. The pre- and post-HD BNP levels were particularly helpful in guiding ultrafiltration goals for each treatment. It not only served as a useful adjunct in the evaluation of the child’s daily fluid status, but also allowed for a stable transition between dialysis modalities. In comparison to the usual markers of fluid status, BNP seemed to correlate well with the child’s volume status and demonstrated a linear decline as fluid removal on CKRT was achieved. The explanation for this trend may be the effect of fluid overload leading to myocardial stretch, dilation of heart chambers, and release of BNP during hypervolemia. Corresponding improvement in BNP was seen when approaching euvolemia and may be due to less myocardial stretch, dilation, and release of BNP from myocytes. To this point, echocardiograms performed at time of respiratory decompensation revealed a dilated left atrium and reduced LV systolic shortening which normalized with normalization of BNP.

**Fig. 5:**
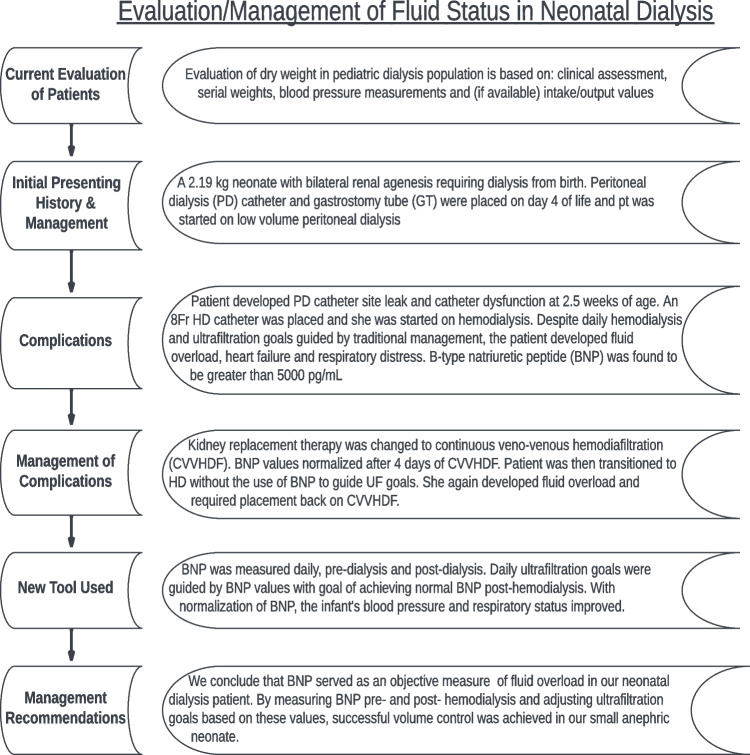
Flowchart summarizing evaluation/management of fluid status in neonatal dialysis

In summary, while the case presentation represents a limited evaluation of a single neonatal patient, the trends in clinical improvement and comparisons to conventionally used markers of fluid balance serve to demonstrate the unique strengths of BNP in the pediatric dialysis population. While traditional markers of fluid status in this population are sometimes difficult to obtain and are known to be potentially problematic, BNP may serve as an additional objective measure of fluid status (Fig. 5). Further research directions may include prospectively enrolling pediatric dialysis patients in a dual arm study evaluating use of BNP in assessment of volume status pre- and post-kidney replacement therapy. With future studies, B-type natriuretic peptide (BNP) may be promoted from perceived cardiac biomarker to a biomarker of volume status in pediatric dialysis patients.

## Data Availability

The data analyzed for this case report are available from the corresponding author on reasonable request.
